# Design of an oral vaccine using *Lactococcus lactis* against brucellosis: an in vitro and in vivo study

**DOI:** 10.1186/s13568-023-01638-4

**Published:** 2024-01-03

**Authors:** Mahsa Kazemi-Roudsari, Abbas Doosti, Mohammad-Saeid Jami

**Affiliations:** 1 Department of Biology, Shahrekord Branch, Islamic Azad University, Shahrekord, Iran; 2 Biotechnology Research Center, Shahrekord Branch, Islamic Azad University, Shahrekord, Iran; 3 Cellular and Molecular Research Center, Basic Health Sciences Research Institute, Shahrekord University of Medical Sciences, Shahrekord, Iran

**Keywords:** Brucellosis, *Lactococcus lactis*, *Omp10*, Oral vaccine

## Abstract

Brucellosis is regarded as one of the world’s most severe zoonotic diseases. This study aimed to investigate the *p*ossibility of using recombinant *Lactococcus lactis* (*L. lactis*) as a live vector to produce recombinant *Brucella abortus* (*B. abortus*) *Omp10*. The gene sequences were obtained from GenBank. The proteins’ immunogenicity was assessed using Vaxijen. After confirming the cloning of the *Omp10* gene in the pNZ8148 vector by enzymatic digestion and PCR, transformation into *L. lactis* was done. SDS-PAGE and western blot methods evaluated *omp10* protein expression. Mice received oral recombinant *L. lactis* vaccines. IgG antibodies against *Omp10* were tested using ELISA. Real-time PCR and ELISA were used to analyze cytokine responses. Survival rate and histopathological changes were evaluated after the challenge. *Omp10* was chosen for its 1.5524 antigenicity score. Enzymatic digestion and PCR identified a 381-bp gene fragment. A 10 kDa band indicated the success of *L. lactis* transformation. Mice administered the *L. lactis*-pNZ8148-*Omp*10-Usp45 vaccination 14 days after priming showed significantly higher *Omp10*-specific total IgG and IgG1 (*P* < 0.001) than the PBS control group. The mice who received the *L. lactis*-pNZ8148-*Omp10*-Usp45 and IRBA vaccines had significantly elevated levels of IFN-γ, TNFα, IL-4, and IL-10 in samples collected on days 14 and 28 (*P* < 0.001). Inflammatory response, morphological damage, alveolar edema, and lymphocyte infiltration were reduced in the target group. A recombinant *L. lactis* expressing the *Omp10* protein was constructed as an oral *Lactococcus*-based vaccine and compared to live attenuated vaccines for future brucellosis investigations.

## Introduction

Brucellosis, sometimes called Malta fever, is a significant global zoonotic disease attributed to Gram-negative bacteria from the genus *Brucella* (Sadeghi et al. 2022). Furthermore, the disease exhibits a substantial annual incidence rate, with over 500,000 new cases reported annually (Shirdast et al. 2021; Fatehi et al. 2023). *Brucella* species, which include *B. melitensis*, *B. abortus*, *B. suis*, *B. canis*, *B. ovis*, and *B. neotomae*, are facultative intracellular bacteria capable of establishing persistent infections within host cells, thus often complicating the process of diagnosis and treatment of diseases (Hou et al. 2019). Live attenuated vaccines (namely *B. melitensis* Rev1 and *B. abortus* RB51) are used for animal immunization as a preventive measure against *Brucella* infection, owing to their significant economic ramifications and medical burden (Hou et al. 2019). The use of live-attenuated vaccinations, despite their influential role in the control of brucellosis in animals, presents some drawbacks, including the excretion of bacteria in milk, potential risks to human health, induction of miscarriage in pregnant animals, and the possible reversion of the attenuated organism to a virulent state. Given these constraints, there remains a constant need to create immunizations displaying high efficacy and safety (Heidary et al. 2022; Darbandi et al. 2022).

Infections might potentially arise after ingesting or inhaling *Brucellae*, which can penetrate mucosal surfaces, including the upper respiratory or gastrointestinal mucosa, via lymphoid cells (Olsen et al. 2022). Infections may arise after ingesting or inhaling *Brucellae*, which can infiltrate mucosal surfaces, including the upper respiratory or gastrointestinal mucosa, via lymphoid cells. After being engulfed by macrophages, dendritic cells (DCs), and other antigen-presenting cells (APCs), it has been shown that about 40–50% of the bacteria exhibit resistance to intracellular digestion (López-Santiago et al. 2019). Given that the mucosal surfaces serve as the primary points of entry for *Brucella* into the human body, it becomes logical to consider the development of a vaccine for brucellosis that may be delivered through the mucosal route (Bialer et al. 2020).

Mucosal surfaces, such as the gastrointestinal, respiratory, and urogenital tracts, are the primary contact points and entrances for many pathogens. Consequently, establishing protective immunity at these mucosal surfaces is often desirable in the realm of novel vaccine development. At present, approved vaccinations for humans or animals are often provided through the parenteral route. However, it is essential to note that vaccines administered via this route usually do not effectively stimulate mucosal immune responses (Huang et al. 2022). In contrast, mucosal-administered vaccines have the potential capacity to elicit both humoral and cell-mediated immune responses at mucosal locations as well as at the systemic level. The combination of mucosal vaccines’ characteristics and their needleless, noninvasive immunization technique makes them an appealing option for vaccination (Skwarczynski and Toth 2020).

There is a growing emphasis on the use of mucosal administration and probiotic-based systems due to the potential of *Lactococcus lactis* (*L. lactis*) to serve as a live vector for expressing viral and bacterial proteins (Diaz-Dinamarca et al. 2020). *L. lactis* is classified as a Gram-positive microorganism characterized by its non-pathogenic and non-invasive nature. It is a member of the *lactic acid bacterium (LAB)* group. Moreover, this organism is commonly recognized as safe (GRAS). Viral, bacterial, and parasite antigens have been successfully produced in *L. lactis*, a bacterium often used in research. These recombinant strains have shown the ability to effectively transfer antigens to the intestines’ mucosal areas, eliciting a targeted immune response (Gouran et al. 2023). Furthermore, studies have shown evidence that *L. lactis* can endure transit through the gastrointestinal system in both animal models and human subjects without establishing colonization (Song et al. 2020). This characteristic makes *L. lactis* a promising contender for developing vaccines for human use. The use of *L. lactis* for immunization offers many benefits owing to the adjuvant qualities of its peptidoglycan wall. Additionally, *L. lactis* may serve as a protein expression system, guaranteeing the production of the antigen (Qiao et al. 2021). Within this particular framework, the use of *Omp10* as an antigen within a strain of *L. lactis* has the potential to function as a vehicle for the delivery of antigens, hence facilitating the creation of an ingestible vaccine. This research aimed to assess the potential of *L. lactis* strains that express *Omp10* to induce a protective humoral and cellular immune response against *Brucella* colonization in a mouse model.

## Materials and methods

### Bacterial strains, culture conditions and plasmid

This study obtained *Escherichia coli* (*E. coli*) Top 10F and *L. lactis* PTCC1336 from the Iran Biological Resources Center. The *L. lactis* bacteria were cultured in M17 broth (DM565) (Quelab, Canada). The bacterium *E. coli* strain Top 10F was cultured in LB broth (Merck, Germany) at 37 °C and a shaking speed of 200 rpm using a shaker incubator. Agar media on the plates were prepared by adding Merck’s microbiological agar medium at a concentration of 1.5%. The *B. abortus* strain 2308 was cultivated in *Brucella* Agar (Himedia, India) with horse serum. The cultivation process was carried out at 37 °C in an environment containing 10% carbon dioxide for 72 h, without any agitation. Brucellosis vaccine IRIBA (IRIBA Vac) was prepared as a positive control. GENEray (GENEray, China) supplied the pNZ8148 *Lactococcus* expression vector.

### Synthesis of recombinant *L. lactis*

The plasmid vector pNZ8148 was synthesized by GENEray company (GENEray, China). The researchers used the Addgene website as a resource to collect information about the nisin-based expression vector pNZ8148. Additionally, the software tool Gene Runner was employed to facilitate the selection of appropriate restriction enzyme cleavage sites. The expression vector pNZ8148 (T) includes several components, notably an origin of replication (ORI), a gene conferring chloramphenicol resistance, two genes encoding replication proteins repA and repC, a nisin-inducible promoter (P nisA), and a transcription terminator. This vector was designed to facilitate the expression of cloned sequences in Gram-positive bacteria, especially LAB. The final construct was synthesized by combining a 381 bp *Omp10* gene fragment, identified as accession number L27995.1, with a 27 amino acid signal peptide sequence named Usp45, associated with accession code ABY84357. The proteins’ immunogenicity and 3D structure were assessed using Vaxijen and SWISS-MODEL. The CELLO program, accessible at http://cello.life.nctu.edu.tw/, was used to ascertain the specific subcellular localization of protein inside the *B. abortus* bacterium. The gene and the signal peptide (Usp45-*Omp10*) were synthetically cloned into the nisin-based vector pNZ8148, specifically between the *Kpn*I and *Xba*I cutting sites (Bohlul et al. 2019).

### Verification of *Omp10* cloning in pNZ8148 vector

The polymerase chain reaction (PCR) was performed to observe the presence of *Omp10* by using primers (Table 1) that specifically target this gene. Furthermore, the cloning process’s precision was confirmed by using enzymatic digestion and sequencing techniques (Rezaei et al. 2020). The PCR process consists of a 2 × 10 mL PCR buffer (amplicons, Denmark), 2 mM of MgCl_2_, 200 μM of dNTPs (Yekta Tajhiz Azma, Iran), 10 pmol of each primer (metabion, Germany), 100 ng of plasmid DNA, and 1 unit of Taq DNA polymerase enzyme (Tajhiz Azma, Iran). The polymerase chain reaction (PCR) temperature protocol comprises an initial annealing phase at 95 °C for 5 min. This is then followed by a series of 30 repeating cycles, each consisting of denaturation at 94 °C for 1 min, annealing at 52 °C for 1 min, and extension at 72 °C for 1 min. The ultimate elongation was ultimately executed at a temperature of 72 °C for 5 min. The PCR product was evaluated using 1% gel electrophoresis. Enzymatic digestion using *Kpn*I and *Xba*I restriction enzymes and determining the nucleotide sequence of the recombinant vector was performed by GENEray.

**Table 1:** The primers used in the present investigation were specifically developed for this research

Gene	Sequence (5′ → 3′)	TM (℃)	Size (bp)
*omp10*	F: 5′-ATGGAAAGTATGGATATGAAAAG-3′	57	381
	R: 5′-TTAACCAGCATTACGACGTGTT-3′		
*GAPDH*	F: 5′-TGTGTCCGTCGTGGATCTGA-3′	60	78
	R: 5′-CCTGCTTCACCACCTTCTTGA-3′		
*IFN-γ*	F:5′-AGCGGCTGACTGAACTCAGATTGTAG-3′	60	199
	F: 5′-GTCACAGTTTTCAGCTGTATAGGG-3′		
*IL-10*	F: 5′-CTTGGGACTGATGCTGGTGAC-3′	60	162
	R: 5′-TCTTTTCTCATTTCCACGATTTC-3′		
*IL-4*	F: 5′-CGAAGAACACCACAGAGAGTGAGCT-3′	60	180
	R: 5′-GACTCATTCATGGTGCAGCTTATCG-3′		
*TNF-α*	F: 5′-AGGCACTCCCCCAAAAGATG′	60	183
	R: 5′-CCACTTGGTGGTTTGTGAGTG-3′		

### Transformation, SDS-PAGE and Western Blot Assay

The *L. lactis* strain was effectively transformed with the recombinant plasmid pNZ8148-Omp10-Usp45 using the electroporation method using a Gene Pulser (Xcell BIO-RAD Gene Pulser; with 2500 V, 25 µF, and 200 Ω). In this experiment, an electroporator introduced genetic material into *L. lactis* host cells. Specifically, 400 μL of the host cells were transfected with 6 μL (0.2 μg/μL) of the recombinant plasmid pNZ8148-Usp45, which carries the *Omp10* gene. Additionally, 6 μL (0.2 μg/μL) of the pNZ8148 vector without the *Omp10* gene was also transfected into the cells as a negative control. A suspension of *L. lactis* bacteria was grown on M17 Agar with 25 μg/mL chloramphenicol. The chloramphenicol-resistant gene in the pNZ8148 plasmid was used as a selection marker to identify the successfully transformed *L. lactis*/pNZ8148-*Omp10*-Usp45 strain (Soumya et al. 2023).

The recombinant *L. lactis* strain comprised pNZ814-Omp10-Usp45 and was cultured on an M17 medium. The media was supplemented with 0.5% glucose, and 25 μg/mL chloramphenicol was added. The recombinant strains were cultured and then induced with a 10 ng/mL nisin concentration until the bacteria reached an optical density of about 0.5 at a OD_600_. The supernatant was collected using centrifugation at 10,000 RPM at 4 °C for 30 min. The resulting bacterial protein supernatants were concentrated 50-fold compared to their initial volume by the salting out method. Subsequently, the concentrated supernatants were analyzed using 12% sodium dodecyl sulfate-polyacrylamide gel electrophoresis (SDS-PAGE). Transferring protein from a gel to a nitrocellulose membrane was conducted, followed by blocking the membrane using a 3% BSA solution. This blocking step included incubating the membrane at 37 °C for 1 h. Following the washing step with washing buffer (PBS/T), the membrane was incubated with Anti-his antibody (HRP conjugated-Rozhan AZMA, Inc., Iran) at a dilution of 1/1000 in PBS. The washing processes were repeated after incubation (1 h, 37 °C). Ultimately, the membrane was subjected to development using diaminobenzidine tetrahydrochloride (DAB) (Liu et al. 2022).

### Animals and immunization

Female BALB/c mice, aged 6 to 8 weeks (weight 25–28 g), were procured from Shahrekord University. The mice were acclimatized and then randomly assigned to different experimental groups. The animals were housed in pathogen-free conditions and had ad libitum access to food and water for the duration of the study. The mice were manipulated and euthanized following the protocols outlined by the Institutional Ethics Committee (IR.IAU.SHK.REC.1401.003). In total, 120 mice were randomly divided into 6 groups in this study, including Group 1: *L. lactis*: pNZ8148-Usp45-*Omp10* has been given to animals through oral vaccination (100 µg of pDNA), Group 2: the group receiving 100 μg of *L. lactis*-pNZ8148 orally, Group 3: *L. lactis* recipient group, Group 4: the group receiving 2 × 10^8^ CFU of IRIBA vaccine subcutaneously, Group 5: the group treated with empty plasmid and Group 6: negative control group (treated with PBS). The standard and transgenic strains of *L. lactis*-pNZ8148-*Omp10* were grown in the same way and induced with nisin for 1 h. A group of mice was given three doses of 10^8^ *L. lactis*-pNZ8148-*Omp10* colony forming unit (CFU) using an oral syringe. The treatment was given over 3 days, from day 0 to day 2, from day 14 to day 16, and from day 28 to day 30.

### Evaluation of cellular immunity

#### Enzyme-linked immunosorbent (ELISA) evaluation of serum cytokine level

To evaluate the level of IFN-γ, TNF-α, IL-10, and IL-4 cytokines, blood samples were collected on days 0, 4, 7, 14, and 28 after immunization. The cellular immunological response induced by oral vaccination was examined by measuring IFN-γ, TNF-α, IL-10, and IL-4 levels. As instructed in the product manual, serum levels of proinflammatory cytokines were measured using Karmania pars gene ELISA kits (KPG, IRAN). Purified r*OMP10*-Usp45 was used to cover the wells of a polycarbonate plate with 100 µL of a 5 µg/mL carbonate buffer (pH 9.6) solution. To mitigate non-specific binding, the plates were subjected to an extra overnight incubation at 4 °C after being washed with a solution of 0.05% (w/v) Tween 20 in PBS. During this incubation, the plates were inhibited with 5% skim milk in PBS containing 0.5% Tween 20. After removing the blocking buffer, plates were incubated with 100 µL of serum samples diluted 1:100 in the blocking buffer for 2 h at room temperature while gently rotated. Goat Anti-Mouse IgG (Bio-Rad Cat No. 170-6516) labeled with HRP was added to the wells at a dilution of 1:2000. After completing the last step of washing, a volume of 50 μL per well of the enzyme–substrate TMB was administered for 30 min at a temperature of 37 °C to quantify the specific reactivity.

#### Real time PCR (qPCR)

Real-time PCR was used to analyze IFN-γ, TNF-α, IL-10, and IL-4 expression levels. The Primer 3 program designed primers specific to the target sequences. Subsequently, the BLAST tool was utilized to confirm the specificity of the produced primers by comparing them against comprehensive gene-bank databases (Table 1). Using an RNeasy microcolumn and the manufacturer’s recommended procedure (Yekta Tajhiz, Iran), total RNA was isolated from 25 to 50 mg of spleen and small intestine tissues. To evaluate the yield and quality of the extracted RNA, the spectrophotometer readings at 260/280 were used to calculate the optical density of the samples. cDNA was synthesized using the First-Aid Reverse Transcription Kit (Fermentas), and the extracted RNA, for q real-time PCR, synthesized cDNA, and selected primers were utilized. The conditions of the reaction were as follows: 95 °C for 15 min, 95 °C for 30 s, 60 °C for 30 s, 72 °C for 30 s in 40 cycles, and one final cycle at 72 °C for 30 s. Every experiment was conducted in triplicate. The GAPDH gene was used as the reference gene to ascertain a normalized value arbitrarily assigned to each gene (Taghiloo et al. 2023).

#### Assessment of humoral immunity

Serum samples were obtained from five mice per group 2 days before each injection and 15 days after the last immunization. The specimen was collected and stored at a temperature of − 70 °C. The identification of serum G1 (IgG1) and secretory IgA (sIgA) from fecal pellets, with specificity to *Omp10*, was accomplished using an indirect ELISA, according to a pre-established protocol. The wells of a polycarbonate plate were coated with 100 μL of a 5 μg/mL solution of pure *Omp10* in carbonate buffer (pH 9.6). After washing with PBS containing 0.05% (w/v) Tween 20, the plates were incubated with 5% skim milk in PBS containing 0.5% Tween 20 for an additional overnight at 4 °C to avoid non-specific binding. After removing the blocking fluid, the plates were incubated with 100 µL of blood samples diluted 1:100 in preventing buffer for 2 h at room temperature while gently rocking. The wells were then incubated at room temperature for an additional hour with a dilution of 1:2000 Goat Anti-Mouse IgG (Bio-Rad Cat No: 170-6516) tagged with HRP. Three further washes with PBS-Tween were performed between incubations. After a final wash, specific reactivity was determined by adding 50 µL/well of the enzyme–substrate TMB and incubating the plates at 37 °C for 30 min. The reaction in each well was stopped by adding 2 M H_2_SO_4_. The optimal density was calculated at 492 nm after 10 min (OD_492_). Each experiment was performed three times.

Mucosal IgA (sIgA) levels were determined by analyzing fecal pellets. Using a balance, we measured and homogenized the fecal pellet samples to a final concentration of 100 mg per 0.5 mL of PBS 1 (pH 7.2) containing 1% BSA and collected them 2 weeks following the last vaccine. The samples were then incubated for 16 h at 4 °C, centrifuged for 5 min at 15,000 RPM at 4 °C, and the supernatants were utilized to detect sIgA.

#### Brucellosis model

In the context of the brucellosis model, BALB/c mice that had been vaccinated were subjected to intravenous infection with 10^4^ CFU of *B. abortus*. Subsequently, their survival was observed over 10 days following the infection. Each group’s body weight, clinical rating, and survival rate were recorded daily. Each mouse’s overall clinical sign was assessed using a sliding scale of 0 to − 5. The animals underwent clinical assessments and were assigned a numerical score based on their health status. A score of 0 indicated that the animals were in a standard, active, and healthy condition. A score of − 1 indicated slight sickness, characterized by slightly ruffled fur but an otherwise standard appearance. A score of − 2 indicated illness, with ruffled fur, sluggish movement, and hunching. A score of − 3 indicated extreme sickness, with ruffled hair, prolonged activity, stooped posture, and closed eyes. A score of − 4 indicated a moribund state, while a score of − 5 indicated that the animal had died. After 2 weeks, the spleens were extracted using sterile techniques, homogenized, and afterward cultured on Blood Agar (QLAB, Spain) to quantify the colony-forming units (CFU) of *B. abortus* per organ.

#### Histological analysis

The spleens were aseptically excised and afterwards immersed in a 10% formalin solution for fixation. Following the process of embedding in paraffin, the obtained tissue slices were subjected to histological examination using a microscope. Subsequently, the slices were stained with hematoxylin and eosin (H&E) for further analysis. An ImagePro macro was used to evaluate spleen damage by determining the fraction of the lesion region relative to the whole spleen area (Ghajari et al. 2021).

### Statistical analyses

The data analysis and statistical testing were conducted using GraphPad Prism 9.0. The study used both one-way and two-way analysis of variance (ANOVA) to assess and compare means. Subsequently, a Tukey–Kramer post hoc test was conducted using a 95 percent confidence interval. The researchers used a Chi-square test with Yates’ correction to assess and compare the survival rates of mice that were inoculated with a specific treatment to those of the control group. Statistical significance was determined at the thresholds of *P* < 0.05 and *P* < 0.01.

## Results

### In-vitro investigation

#### pNZ8148-Usp45-*Omp10* recombinant DNA sequence verification

**Fig. 1:**
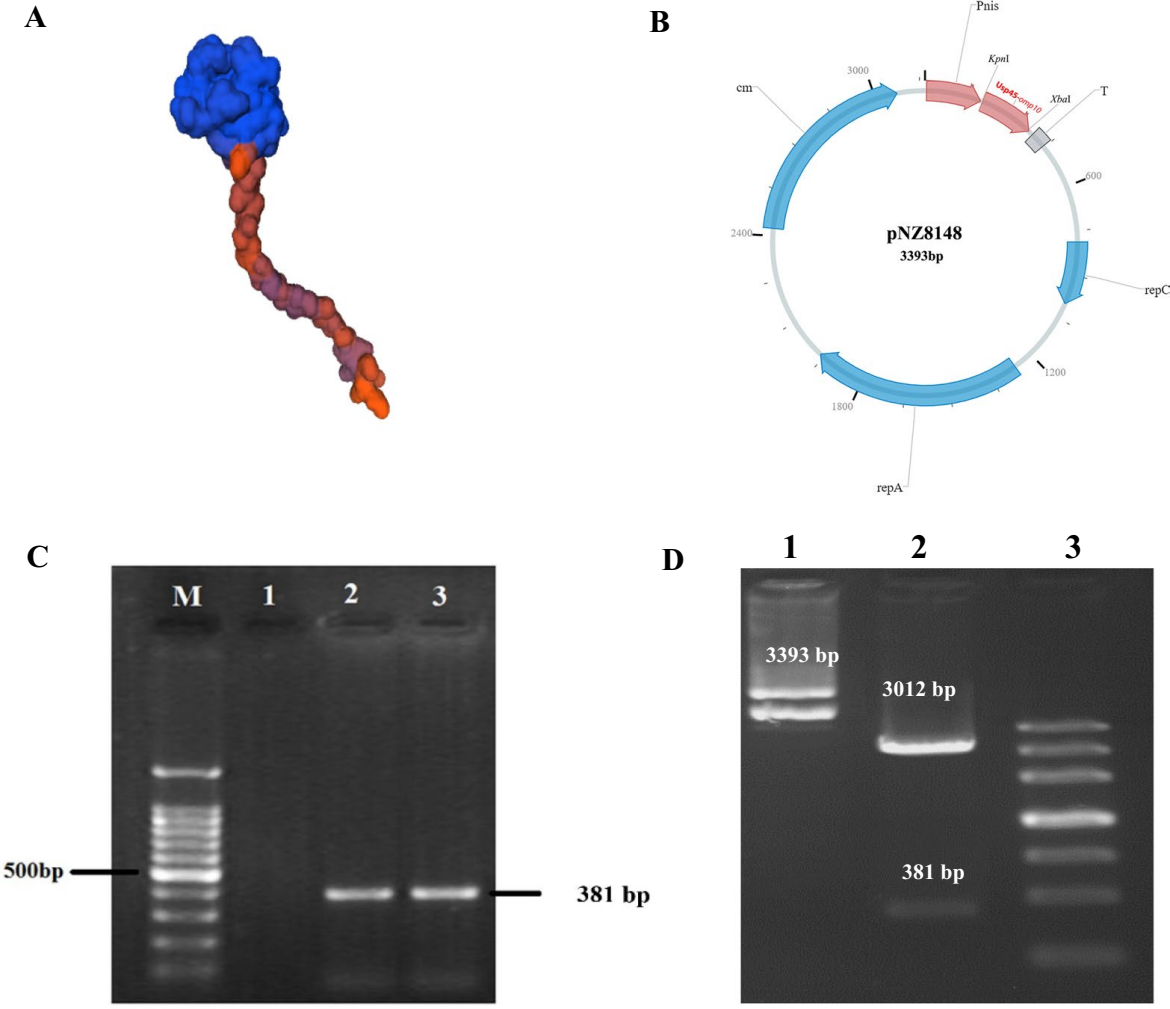
**A** 3D structure of *Omp10* protein obtained from SWISS-MODEL online software. **B** pNZ8148-Usp45-*Omp10* vector gene map with a length of 3393 bp. **C** Observation of 381 bp gene fragment belonging to *Omp10* gene in 1% agarose gel to confirm cloning (Lane 2 and 3), Lane 1: negative control, M: marker 100 bp (Parstous, Iran). **D** Lane 1: recombinant plasmid prior to enzyme digestion, Lane 2: enzymatic digestion and observation of 381 bp gene fragment belonging to *Omp10* and 3012 bp gene fragment belonging to pNZ8148 vector. Lane 3: 1 kb DNA ladder (GENEON, Germany)

The antigenicity of the selected protein sequences was evaluated using the VaxiJen in-silico tool, with a cut-off value of 0.5. The antigen *Omp10* was chosen because of its favorable antigenicity score of 1.5524. Also, the 3D structure of the desired protein was designed using SWISS-MODEL online tool (Fig. 1A). Using CELLO software, the exact location of *Omp10* protein was determined. This protein was determined outer membrane and with reliability 1.079. Figure 1B displays the schematic representation of the pNZ8148-Usp45-*Omp10* plasmid, as generated using Snap Gene software. The final length of this recombinant vector is 3393 bp. The verification of the recombinant plasmid pNZ8148-Usp45-*Omp10* was conducted by the use of direct colony PCR and double digestion analysis using two specific restriction enzymes. The presence of the 381 bp band confirmed that the *Omp10* gene had been successfully cloned into the pNZ8148 vector (Fig. 1C). The precision of the cloning procedure was also verified by using *Kpn*I and *Xba*I double digestion. The association between the existence of a 3081 bp band and 381 bp amplicon belonging to the *Omp10* gene was observed (Fig. 1D). The examination of the findings obtained from the sequencing of recombinant plasmids was conducted using the Blast algorithm. The analysis of the blast results indicated that the recombinant plasmids had a significant similarity to the target bacteria, with an E-value of 8e−94, a query cover of 100%, and a Per. Ident of 99.52.

#### Examining the expression of the recombinant *L. lactis* /pNZ8148-*Omp10*-Usp45

**Fig. 2:**
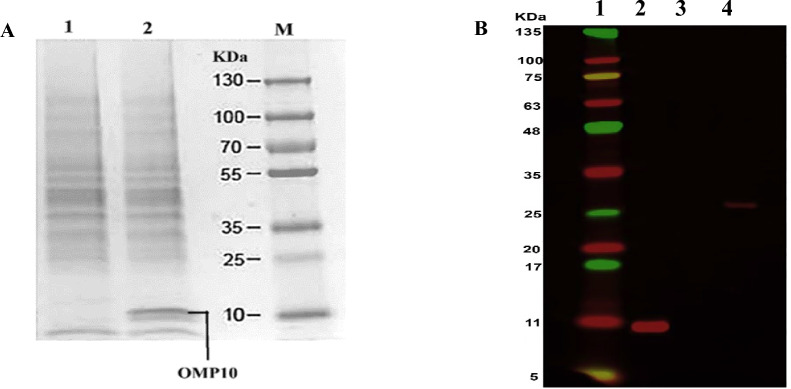
**A** Electrophoresis of the recombinant *L. lactis* protein mixture using SDS-PAGE. In lane 2, *L. lactis* bacteria were transformed with the recombinant *Omp10* vector pNZ8148-Usp45. Lane 1: a vector that does not express the desired protein was used to transform *L. lactis* bacteria. Lane M: indicator (Thermo Fisher Scientific, USA). **B** To confirm the existence of recombinant protein inside *L. lactis* bacteria that have undergone transformation, a western blot analysis was conducted. Lane 1: marker ((Thermo Fisher Scientific, USA), Lane 2: the *L. lactis* bacteria were subjected to transformation using the recombinant vector pNZ8148-*Omp10*-Usp45. Lane 3: *L. lactis* bacteria were transformed using a vector that did not include the gene of interest. Lane 4: GAPDH

The analysis of *Omp10* protein expression in *L. lactis*/pNZ8148-*Omp10*-Usp45 was conducted using a 12% SDS-PAGE gel (Fig. 2A). The coding region of the fusion *Omp10* gene has a length of 381 bp, and the resulting band seen in the experimental analysis occurred at around 10 KDa. Western blot analysis provided more evidence of protein production at the protein level. Figure 2B displays the Western blot analysis of *L. lactis* protein samples derived from our recombinant *L. lactis*/pNZ8148-*Omp10*-Usp45 organism, as well as the bacteria that underwent transformation using an empty vector (pNZ8148).

### In vivo analysis

#### Assessment of humoral immunity

**Fig. 3:**
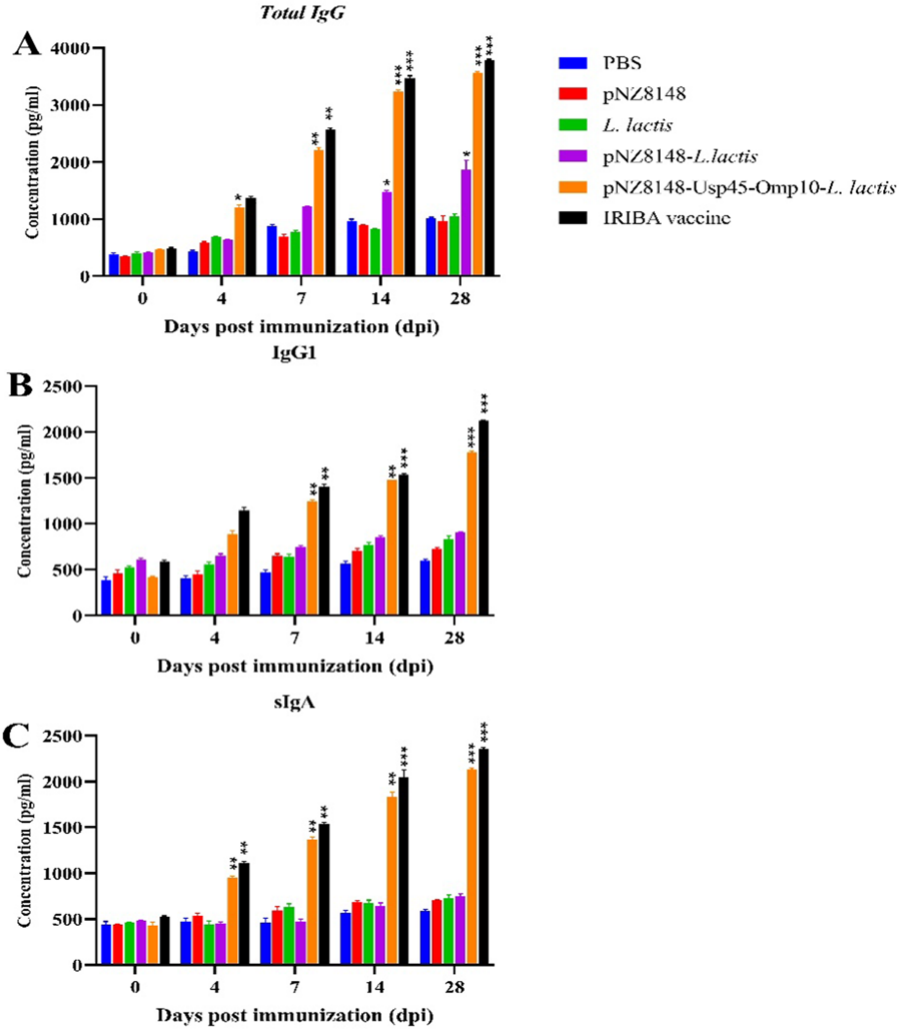
Investigation of humoral immunity after vaccination with different formulations. **A** The study investigates the presence of *Omp10*-specific mucosal total IgG antibodies in mice. The sample was collected from mice after oral vaccination with several vaccine groups. **B** After a period of 30 days after the administration of the last vaccine, BALB/c mice demonstrate a humoral immune response. Serum dilutions of *Omp10*-specific IgG1 from different groups of mice were prepared at a ratio of 1:200. **C** The presence of *Omp10*-specific mucosal secretory IgA (sIgA) antibodies was detected in fecal pellets. Data are presented as mean ± SD. *P < 0.05, **P < 0.01, ***P < 0.001, ****P < 0.0001

A group of 20 female BALB/c mice, aged 6 weeks, were subjected to immunization using the *L. lactis*-pNZ8148-*Omp10*-Usp45 through oral injection, following the procedures outlined in the Materials and Methods section. The mice were vaccinated with 100 μg of the *L. lactis*-pNZ8148-*Omp10*-Usp45 at intervals of every 8 h for 3 days. The serum samples obtained 15 days after the last vaccination were subjected to analysis to determine the levels of antigen-specific antibodies using the indirect ELISA technique. As expected, the presence of *Omp10*-specific antibodies was not seen in the sera of mice vaccinated with phosphate-buffered saline (PBS). The examination of the IgG response revealed a notable augmentation when compared to the control group that received PBS. Furthermore, the alterations in the IgG antibody response within the cohort immunized with intragastric *L. lactis*-pNZ8148-*Omp10-*Usp45 were observed to be significantly distinct both before and after the final vaccination (Fig. 3A). As seen in Fig. 3A, B following a duration of 28 days, it was observed that the group supplied with PBS did not exhibit any noteworthy production of total IgG and IgG1 antibodies (*P* > 0.05). The *L. lactis*-pNZ8148-*Omp10*-Usp45 group exhibited significantly higher levels of serum total IgG and IgG1 titers compared to the PBS and pNZ814 groups (*P* < 0.05). The serum IgG antibody values in the group that received *L. lactis*-pNZ8148 were marginally higher compared to the group that received *L. lactis* alone. However, these differences did not reach statistical significance (*P* > 0.05) and were lower than the differences observed in the groups that received *L. lactis*-pNZ8148-*Omp10*-Usp45 and IRIBA-vac (*P* < 0.001). Additionally, we evaluated the occurrence of mucosal responses in animals that had been administered vaccines. The value of *Omp10*-specific secretory immunoglobulin A (sIgA) was quantified. Based on the results obtained, it was shown that mice who received *L. lactis*-pNZ8148-*Omp10*-Usp45 vaccination exhibited a higher production of antigen-specific mucosal IgA compared to control mice treated with PBS or other experimental groups (*P* < 0.05) (Fig. 3C).

#### Measurement of serum levels of cytokines

**Fig. 4:**
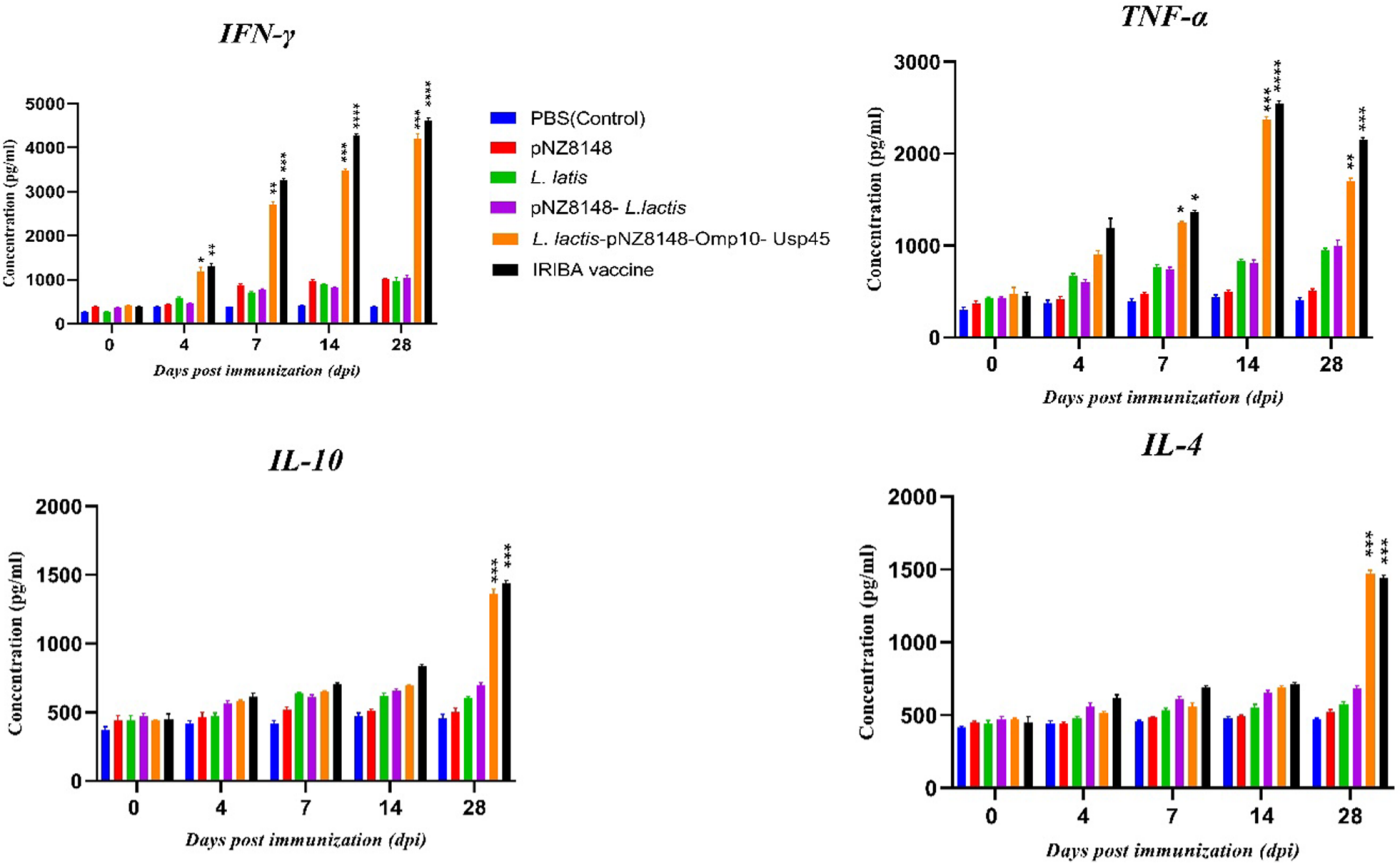
Measurement of serum levels of IFN-γ, TNF-α, IL-10 and IL-4 cytokines by ELISA method. *P < 0.05, **P < 0.01, ***P < 0.001, ****P < 0.0001

The ELISA technique evaluated serum levels of IFN-γ, TNF-α, IL-10, and IL-4 cytokines on days 0, 4, 7, 14, and 28 after vaccination. The results obtained from this study showed that the serum levels of IFN-γ, TNF-α, IL-10, and IL-4 in the groups vaccinated with empty plasmid, *L. lactis*, *L. lactis*-pNZ8148 on different days of vaccination were not significantly different from the control group (Fig. 4). On the other hand, the results showed that the serum level of IFN-γ and TNF-α reached its peak on the 14th and 28th days after vaccination with *L. lactis*-pNZ8148-*Omp10*-Usp45 and IRIBA, and it was significantly different from the PBS group (*P* < 0.0001) (Fig. 4). On day 28, despite a decrease compared to controls, IFN-γ concentration remained significantly higher. IL-4 and IL-10 induction was assessed in mice that had been administered immunizations. The results indicated no production of IL-4 or IL-10 was seen in mice who received the vaccination, even after 14 days post-immunization. Also, the results showed an increase in the production of IL-10 and IL-4 in the groups vaccinated with IRIBA and *L. lactis*-pNZ8148-*Omp10*-Usp45 on the 28th day after vaccination (*P* < 0.001).

#### Assessment of cell immunity by q-PCR in spleen and small intestine

**Fig. 5:**
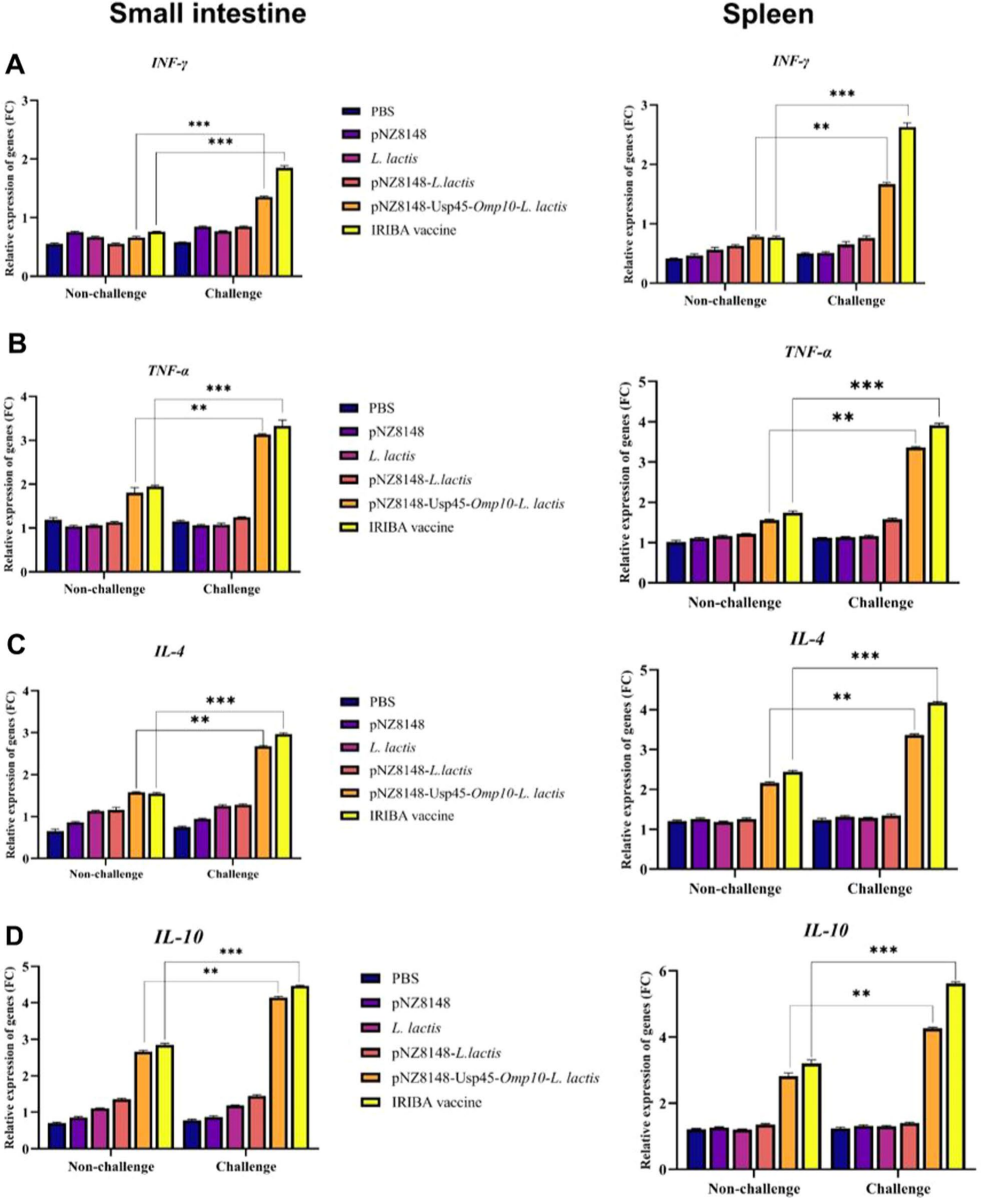
Measurement of the secretion level of IFN-γ, TNF-α, IL-4 and IL-10 cytokines in the small intestine and spleen by real-time PCR method. **A** Expression levels of IFN-γ cytokine in spleen and small intestine before and after challenge with *B. abortus*, **B** measurement of TNF-α levels before and after challenge, **C**, **D** measurement of IL-10 and 1L-4 expression levels in spleen and small intestine before and after challenge. The data are replicated three times and averaged. *P < 0.05, **P < 0.01, ***P < 0.001, ****P < 0.0001

The secretion of IFN-γ and TNF-α, IL-4, and IL-10 cytokines, which are the main characteristics of cellular immunity, was evaluated in vaccinated and non-vaccinated mice before and after the challenge by q-PCR method at the spleen and small intestine (Fig. 5). The results showed that IFN-γ and TNF-α cytokines expression in the groups vaccinated with *L. lactis*-pNZ8148-*Omp10*-Usp45 and IRBA significantly differed from other groups (Fig. 5A, B). In contrast, the cells derived from non-immunized animals treated with PBS did not exhibit a statistically significant increase in their production of IFN-γ, TNF-α, IL-4, and IL-10 upon restimulation. The *L. lactis*-pNZ8148-Usp45-*omp10* and IRBA vaccination groups had significantly greater IFN-γ and TNF-α transcription levels than others. In addition, the induction of IL-4 and IL-10 by immunized animals was evaluated (Fig. 5C, D). The results showed that the expression level of these cytokines in the spleen and small intestine in the groups vaccinated with IRIBA and *L. lactis*-pNZ8148-Usp45-*omp10* increased significantly after the challenge. Mice that received the IRIBA immunization had substantially higher levels of IL-4 and IL-10 titers 28 days after vaccination (*P* < 0.05) compared to mice in the control groups exposed to the pathogen. Mice vaccinated with empty plasmid, *L. lactis* and *L. lactis*-pNZ8148 and plasmid showed no significant difference in the expression of these cytokines compared to the control group.

#### The survival rate, bacterial loads, body weight changes, and clinical scores of mice post-challenge

**Fig. 6:**
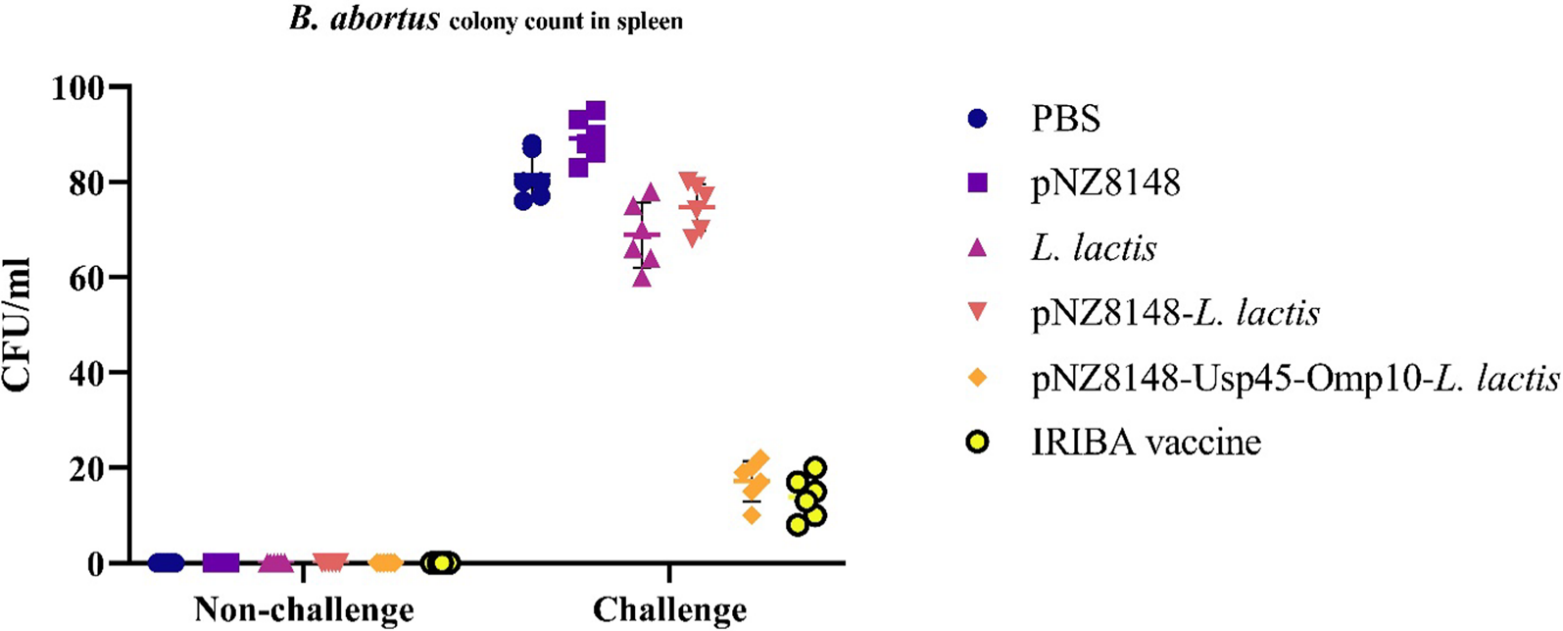
The bacterial loads present in the spleen tissues of BALB/c mice 2 weeks post-challenge

To assess the efficacy of the *L. lactis*-pNZ8148–Usp45–*Omp10* vaccination, we conducted a rigorous experiment in BALB/c mice, using a demanding methodology to measure the level of protection achieved. In this method, all groups under study were challenged with 10^4^ CFU of *B. abortus* after vaccination. Spleen weight of mice that received *L. lactis*-pNZ8148-*Omp10*-Usp45 and IRIBA was significantly lower after 10 days of infection compared to mice in PBS, *L. lactis*, pNZ8148, *L. lactis*-pNZ814 groups. The bacterial load in the groups immunized with IRIBA, and *L. lactis*-pNZ8148-*Omp10*-Usp45 was very low and did not change significantly compared to the non-challenged group (Fig. 6). The daily deaths, weight changes, and overall health of six randomly selected *B. abortus*-exposed mice were monitored for 10 days. Table 2 reveals all of the animals in the PBS and free pNZ8148 groups died. The survival rates of mouse models that were vaccinated with *L. lactis*-pNZ8148-*Omp10*-Usp45 and then exposed to a fatal dose of *B. abortus* isolates were found to be 83.33% over 10 days (Table 2). These values were close to those of mice immunized with IRIBA vaccine (100%). Following exposure to *B. abortus*, each group’s body mass and clinical symptom ratings achieved their minimum values during 2 to 3 days. The body weight of the mice reverted to the mean value within a week after the experiment, and the symptoms gradually diminished.

**Table 2:** Survival percentage, spleen weight and clinical indicators of each group

Groups	Before challenge			After challenge			
	Body weight	Spleen weight	Clinical score	Body weight	Spleen weight	Clinical score	Survival rate (%)
PBS	22.42	415	0	17.23	687	− 5	0
Free pNZ8148	23.84	398	0	20.85	723	− 4	0
*L. lactis*	20.71	423	0	18.56	845	− 3	16.66
*L. lactis*-pNZ8148	24.04	409	0	19.23	654	− 2	33.33
*L. lactis*-pNZ8148-*Omp10*-Usp45	25.32	418	0	22.67	478	0	83.33
IRIBA vaccine	22.78	394	0	19.23	432	0	100

0 (normal, active, healthy), − 1 (slightly sick, slightly ruffled fur, otherwise normal), − 2 (ill, ruffled fur, sluggish movement, hunching), − 3 (extremely sick, ruffled hair, very slow movement, stooped, eyes shut), − 4 (moribund), and − 5 (dead)

#### Pathological changes

**Fig. 7:**
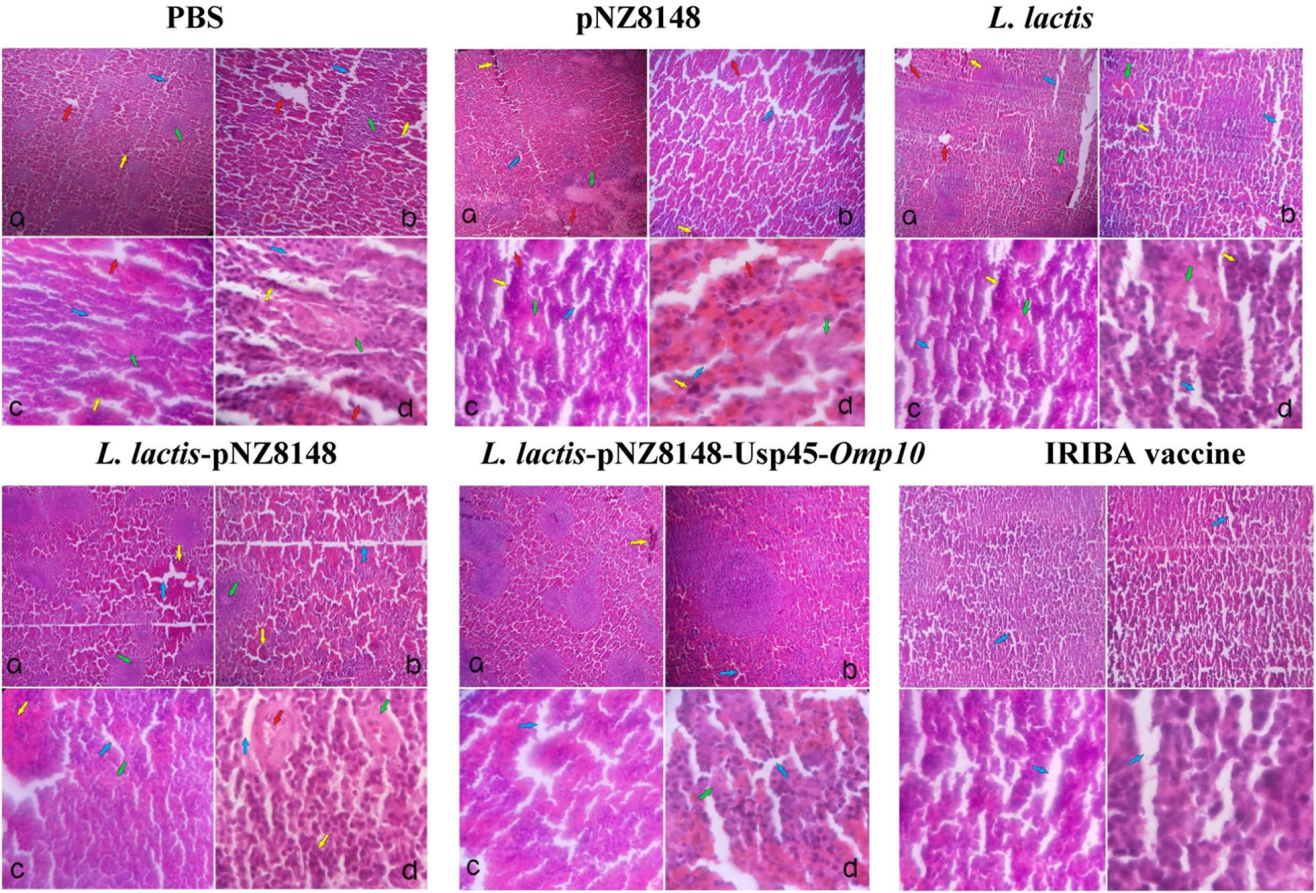
Hematoxylin & eosin staining of spleen tissue sections. **a**–**d** Respectively magnification ×4, ×10, ×40, ×100—increased space of sinusoids (blue arrow), spleen tissue necrosis (green arrow), bleeding in the tissue (yellow arrow), abscess in the tissue, brucellosis (red arrow)

Following exposure to virulent *B. abortus*, the spleens of the mice that were administered with PBS, *L. lactis*, *L. lactis*-pNZ8148, pNZ8148, and *L. lactis*-pNZ8148-Usp45-*Omp10* were obtained. These spleens were then preserved in a 10% neutral buffered formalin solution and processed for H&E staining. According to the findings shown in Fig. 7, it can be seen that the group treated with *L. lactis*-pNZ8148-*Omp10*-Usp45 and IRIBA vaccine had fewer signs of spleen damage, alveolar edema, lymphocyte infiltration, and topological damage caused by the inflammatory process, in comparison to the other groups. The vaccination groups of *L. lactis*-pNZ8148-*Omp10*-Usp45 demonstrated improved alveolar cleanliness and exhibited a higher degree of normalcy than the other groups. The severity of spleen failure in the groups treated with *L. lactis*-pNZ8148-*Omp10*-Usp45 was significantly lower than the other groups after immunization, suggesting that the animals exhibited a less inflammatory response to spleen failure.

## Discussion

The prevalence of brucellosis in the Middle East and its significant economic impact on the cattle business necessitates the rigorous recommendation of vaccination as a preventive measure against this disease (Dorneles et al. 2015). Novel vaccination approaches that prioritize the establishment of robust immune responses, reduction of adverse effects, safe handling procedures, uncomplicated administration, and cost-effective production and distribution have the potential to address safety concerns and mitigate the drawbacks associated with the current utilization of live attenuated *Brucella* strains for brucellosis control (Hou et al. 2019). The efficacy of vaccination strategies lies in identifying and using novel immunogenic antigens of *Brucella*, which may be employed in the manufacture of recombinant vaccines. Limited studies have been conducted on the use of *E. coli* for the production of a recombinant vaccine aimed at preventing brucellosis (Gupta et al. 2012). Many researchers have regarded live vector vaccination systems using probiotics as a proficient method for delivering antigens. The LAB has been widely used as a reliable model in several research investigations (Qiao et al. 2021). *L. lactis*, a Generally Regarded As Safe (GRAS) Gram-positive LAB, has been used to manufacture food for thousands of years (Levit et al. 2022). Today, however, it serves as a cell factory for creating heterologous proteins for medicinal and commercial applications, providing a novel and exciting option for the manufacture of proteins at an industrial scale (Levit et al. 2022). The primary objective of this work is to demonstrate *L. lactis* as a viable option for developing safe and non-pathogenic mucosal live vaccines. Additionally, the study aims to explore the potential of producing the immunogenic *Omp10* protein as an alternative machinery system to *E. coli*, considering its advantageous characteristics. In this study, the *Omp10* gene fragment with a length of 381 bp, along with Usp45, the signal peptide was cloned in pNZ8148 vector by *Kpn*I and *Xba*I restriction enzymes. This is the first study to investigate the cloning, production, and purification of a fusion protein, including *Omp10* and Usp45. The results obtained from PCR, enzyme digestion, and sequencing indicated the successful cloning of *Omp10*-Usp45 in the pNZ8148 vector. Using Western blotting and SDS-PAGE analysis, we demonstrated that *L. lactis efficiently secreted Omp10*.

To ensure its intracellular survival, *Brucella* has developed many strategies to evade host detection and successfully establish infection. After bacteremia, *Brucella* specifically targets macrophages as one of the primary cells to maintain condition. After establishing intracellular infection, *brucellae* exhibit enhanced resistance to removal due to their possession of many mechanisms that enable evasion of the host immune system (Pascual et al. 2022). Therefore, it can be seen that *brucellae* are present inside vacuoles that contain *Brucella* (Pascual et al. 2022). The presence of cellular immunity is crucial in providing defense against brucellosis. The eradication of *brucellae* necessitates an inflammatory or T helper (Th)1 cell response, contingent upon the presence of IL-12 and TNF-α, and is responsible for the induction of IFN-γ (Yingst and Hoover 2003). Brucellosis exhibits systemic dissemination irrespective of the mode of exposure. One notable benefit of mucosal vaccination is its ability to provide localized protection to the mucosal surfaces near infection sites, hence inducing the activation of memory T cells that effectively hinder subsequent reinfection (Pascual et al. 2022). In addition to eliciting a response from the mucosal immune system, mucosal vaccination also leads to the development of systemic immunity (Pascual et al. 2022). One significant benefit of mucosal vaccination is its ability to provide protective immunity in both mucosal and systemic organs. Indeed, the acquisition of *Brucella* infection occurs due to mucosal exposure. Currently, immunization is a very successful strategy for preventing and managing animal brucellosis (Li et al. 2022). When administered to animals, the optimal vaccine for brucellosis should possess avirulent or attenuated characteristics, effectively preventing *Brucella* infections. Simultaneously, it should not impede the accuracy of serological diagnostic methods (Heidary et al. 2022). Furthermore, the intervention must have the capability to effectively inhibit abortion and virulence reversal while concurrently facilitating extended durations of immune protection (Li et al. 2022). Nevertheless, contemporary vaccinations have significant drawbacks that restrict their use, notwithstanding their efficacy in averting animal infections. Hence, the endeavor to create a vaccination that is both safe and productive is a challenging undertaking. Outer membrane proteins (OMPs) are a subset of surface antigens found in *Brucella* (Li et al. 2022; Heidary et al. 2022). One may argue that oligomeric membrane proteins (OMPs) possess favorable attributes that provide viable options for producing recombinant proteins intended for both preventive and diagnostic purposes (Heidary et al. 2022). These attributes include their safeguarded sequences, antigenic properties, and elevated pathogenicity levels. OMPs have been shown to have a significant role in activating both cellular and humoral immunity (Ducrotoy et al. 2016). These proteins have been identified as crucial antigens with protective properties and the capacity to induce an immune response. Our study successfully showed that the administration of live *L. lactis* expressing *Omp10* by oral vaccination in mice resulted in a noteworthy increase in the production of *Omp10*-specific mucosal IgA antibodies. Villena et al. (2008) conducted a study whereby *rL. lactis* was used as a vehicle for the presentation of pneumococcal protective protein A (PppA). Immunological investigations have further shown that the oral administration of the vaccine led to a significant increase in serum and mucosal IgA (Villena et al. 2008). Our research also found that the IgA level in fecal pellets of mice immunized with live *L. lactis*-pNZ8148-Usp45-*Omp10* and IRIBA vaccine was much higher than that of the control group. Our findings were consistent with those of two earlier investigations that used *rL. lactis* as a delivery method for SOD and L7/L12 antigens from *B. abortus* and found similar levels of antigen-specific IgA production (Sáez et al. 2012; Pontes et al. 2003).

During *Brucella* infection, the host’s immune response includes both cellular and humoral immunity (Perkins et al. 2010). The generation of IgG2a antibodies distinguishes the former condition, whereas the latter condition is determined by the generation of IgG1 antibodies. The involvement of IgG1 and IgG2a antibodies in the phagocytic elimination of *Brucella* by macrophages is important (Perkins et al. 2010). BALB/c mice were vaccinated with several agents, including PBS, *L. lactis*, pNZ8148, *L. lactis*-pNZ8148, and *L. lactis*-pNZ8148-Usp45-*Omp10*, to investigate the effect on humoral immunity. Mice serum was collected at 0, 4, 7, 14, and 28 days post-immunization. The results showed a significant increase in total IgG and IgG1 antibody titers on days 14 and 28 after vaccination with *L. lactis*-pNZ8148-Usp45-*Omp10* and IRIBA vaccine, While the titer of antibodies in other groups did not show a significant increase. In addition, the level of cytokines IFN-γ, TNF-α, IL10, and IL-4 as markers of cellular immunity was investigated by ELISA and qPCR. IFN-γ can induce Th1 cell differentiation, resulting in the generation of IgG2a antibodies by activated plasma cells. IFN-γ is necessary for the expression of macrophage bactericidal activity, as it stimulates the activation of macrophages, resulting in an increased ability to eliminate bacteria and suppress microbial proliferation (Priyanka et al. 2021). IFN-γ assumes a crucial function in eliminating intracellular *Brucella* pathogens in the first phases of infection (Priyanka et al. 2021). In the current investigation, it was shown that the expression of IFN-γ was significantly elevated in mice vaccinated with *L. lactis*-pNZ8148-Usp45-*Omp10* and IRIBA vaccine on both day 14 and day 28, as compared to the control group. TNF-α is recognized as a crucial mediator in the immune response of macrophages against *Brucella* infection. TNF-α plays a crucial role in facilitating the recruitment of phagocytes to the site of infection and activating macrophages (Sağmak Tartar et al. 2019). Additionally, TNF-α is of utmost significance in orchestrating immune responses against intracellular infections (Sağmak Tartar et al. 2019). This study’s findings indicate an increase in the production of proinflammatory cytokines, such as TNF-α, in both the *L. lactis*-pNZ8148-Usp45-*Omp10* and IRIBA groups. In contrast, the cytokines IL-10 and IL-4 can suppress the activity of macrophages, enhancing the host’s resistance to *Brucella* infection. The findings of the current research indicate that mice inoculated with *L. lactis*-pNZ8148-Usp45-*Omp10* and IRIB exhibited reduced levels of IL-10 and IL-14 production in comparison to the control group, namely on days 7 and 14 post-vaccination.

The optimal vaccination should induce more protection even in the absence of virulence for the host. We discovered that *L. lactis*-pNZ8148-Usp45-*Omp10* and IRIBA vaccines protect against *B. abortus* in BALB/c. The administration of pNZ8148-Usp45-*Omp10* resulted in a notable degree of immunity compared to the control groups receiving PBS and the free vector. This investigation showed that the live-attenuated vaccine (IRIBA) protected significantly against *Brucella* infection. However, it is worth noting that the difference in protection between mice inoculated with *L. lactis*-pNZ8148-Usp45-*Omp10* and IRIBA was not statistically significant. In the present case, a noteworthy decrease in bacterial load has been seen in the mice administered with *L. lactis*-pNZ8148-Usp45-*Omp10*, indicating that a live vector vaccine can potentially mitigate bacterial colonization. Previous studies have elucidated the inherent characteristics of live vector vaccines in inducing a specific immune response against antigens associated with various illnesses (Diaz-Dinamarca et al. 2020; Gouran et al. 2023). The results of this study indicate that the use of *L. lactis*-pNZ8148-Usp45-*Omp10* as a means of expressing vectors has the potential to provide cost-effective, easily administered, and efficacious live oral vaccines for the prevention of *B. abortus* infection. Serum antibodies are paramount in protecting mucosal surfaces due to their opsonophagocytic activity. As a result, our study offers a novel method for using the food-grade, nonpathogenic, and noncommercial bacterium *L. lactis* as a protein cell factory to produce the novel immunogenic fusion candidate r*Omp10*. This method offers an appealing new approach to assessing the cost-effective, safe, sustainable, simple pilot development of pharmaceutical products. It suggests a workable method for delivering additional heterologous protein antigens. We have already started doing further studies in our labs, such as testing the immunogenicity and protective effectiveness of this recombinant *L. lactis*-r*Omp10* strain as a potential oral vaccine candidate versus virulent *Brucella* infection challenge in mice. This study constructed a live *L. lactis* vaccine expressing *omp10*, which was effective as an oral vaccine in BALB/C mice. The recombinant strain vaccine induced both humoral and cellular responses and improved the immune system compared to the control groups.

## Data Availability

The data generated or analyzed during this study are included in this article and its additional materials.
